# Consumer Acceptance of Texture-Modified Mackerel Stew Products in Older Adults

**DOI:** 10.3390/foods12224049

**Published:** 2023-11-07

**Authors:** Hye-Ji Seo, Seo-Jin Chung, Mi-Sook Cho, Ju-Yeon Park, Jieun Oh

**Affiliations:** 1 Department of Nutritional Science and Food Management, Ewha Womans University, Seoul 03760, Republic of Korea; hyejiseo@ewhain.net (H.-J.S.); sc79d@ewha.ac.kr (S.-J.C.); misocho@ewha.ac.kr (M.-S.C.); 2 Hyundai Green Food. Co., Yongin 16827, Republic of Korea; 3 College of Science & Industry Convergence, Ewha Womans University, Seoul 03760, Republic of Korea

**Keywords:** consumer acceptance, senior-friendly food, texture-modified technology, Atlantic mackerel

## Abstract

Along with concerns regarding societal aging, the dietary requirements of older adults have become a priority. Older adults in Korea experience difficulties consuming animal protein sources as they age. Therefore, this study aimed to develop a senior-friendly food product using mackerel. Accordingly, carbohydrates and proteins were added to the brine solution before saturated vapor treatment. Calcium lactate and poly-gamma-glutamic acid were added to the sauce, and when compared to four commercial products (GT_R, GT_K, PC_K, and AC_G) in an acceptance test, the product was found to exhibit the highest overall liking score (*p* < 0.001). Higher flavor-liking and familiarity ratings were found to increase purchase intention, while higher flavor-liking, overall-liking, and familiarity ratings increased recommendation intention. Those in mid-to-late adulthood preferred the GT_R and PC_K samples, whereas the AC_G sample was preferred by those in very late adulthood. AC_G sample analysis suggested that those in the very late adulthood group had a relatively higher acceptance of spiciness. In this study, a calcium-added mackerel stew product was manufactured, meeting the standards for senior-friendly food in Korea. It will serve as a baseline for further research on fish- and mackerel-based foods for older adults, which is in its early stages.

## 1. Introduction

The older population is growing rapidly worldwide [1], and in Korea, the proportion of older people aged ≥65 years, which was 17.5% of the total population in 2022, is expected to exceed 40% by 2050 [2]. Along with concerns regarding societal aging, the gravity of nutritional and dietary challenges among older adults is also emerging. In addition to reduced intake and poor nutritional status, impaired chewing and swallowing abilities can increase the risk of complications such as malnutrition and aspiration pneumonia [3,4,5,6]. 

The consumption of animal protein sources can be challenging, especially with advancing age and in individuals with chewing and swallowing difficulties. Although animal protein is substantially bioavailable and effective in preventing sarcopenia [7,8,9], older adults in Korea have been shown to acquire only one-third of their total protein from animal sources [10,11]. This is significantly lower than that in Western seniors, who derive >60% of their protein from animal sources [12,13,14], suggesting the need to develop animal protein-source foods that ensure adequate protein intake among Korean seniors. Fish is a representative protein-source food, and mackerel is the most preferred seafood among Koreans [15]. The Atlantic mackerel (*Scomber scombrus*), a widely distributed pelagic and migratory fish, is a valuable source of protein and omega-3 fatty acids [16,17]. According to the Food and Agriculture Organization of the United Nations (FAO) report, it has received increasing attention because of its growing catch and economic importance [18]. Most studies on mackerel-based senior-friendly foods have used reformulation technology [19,20,21], which may be unfamiliar to consumers and is limited by its low acceptance; therefore, developing products that retain their original shape by considering sensory factors is imperative.

Marination entails the immersion of meat products in a brine solution to improve their quality, thus increasing their juiciness, salinity, and hydration [22,23]. Salt and phosphate are commonly used, and additional materials can be added to the brine solution to increase its softening effect [24,25]. Both saturated vapor and marination, which control hardness while maintaining the food’s original shape, are suitable for producing senior-friendly foods, and studies that have measured the softening effect of parallel treatment include those by Jang and Ahn [26,27]. However, no studies have examined the effects of softening materials and saturated steam technology on fish. In addition, phosphates have been reported to decrease intestinal mineral absorption, potentially increasing the risk of bone-related diseases and increasing the risk of chronic kidney disease when consumed in excess; thus, alternative materials are currently being explored [28,29,30,31,32]. 

Among the ingredients used to improve the texture of meat products, carbohydrate ingredients, such as sugars and sugar alcohols, are used to increase the water-holding capacity and exert a softening effect [33,34,35], while proteins are used to enhance the nutritional value and increase the water-holding capacity and stability [36,37,38]. Xylose is a highly reactive pentose that is used to enhance the flavor and color of foods by promoting the Maillard reaction through heating [39,40], and the water retention and stability of 3D-printed meat analogs improves as the amount of xylose increases [41]. Soy protein is one of the most available protein sources for good texture properties, including soy protein isolates and soy protein concentrates [25,42]. Soy protein concentrates have fat and water holding properties, modifying the food viscosity and texture [43]. Xylose and soy protein concentrates were selected after preliminary experiments for their ability to soften foods and reduce unappealing odors. Therefore, this study aimed to minimize the phosphate content and confirm the softening effect of carbohydrates and proteins by using xylose and soy protein concentrates in the brine solution with saturated steam in a parallel treatment.

Calcium is an essential nutrient for sustaining life and preventing osteoporosis [44,45]. Older adults are recommended to consume at least 1000–1200 mg/day of calcium for bone health. However, the average dietary intake in Western countries is 700–900 mg/day, and it is even lower in Africa and Asia [46]. Further, the proportions of men and women in Korea meeting the recommended daily allowance (RDA) for calcium has been shown to decrease with age [47]. Therefore, this study aimed to develop a senior-friendly food product that caters to the physical characteristics and various preferences of older consumers. This was achieved by manufacturing texture-modified stewed mackerel by applying a saturated vapor treatment and softening materials, and by utilizing a calcium-fortified sauce. Sensory acceptance was investigated among older people to compare consumer preference for our product with four similar products sold in the market and to verify our product’s commercialization potential.

## 2. Materials and Methods

### 2.1. Materials

The frozen and trimmed Norwegian mackerel (*Scomber scombrus*) used in this experiment were purchased from Sang Won Fisheries (Hanam, Republic of Korea) in February 2023. The following ingredients were included in the brine: purified water (Joylife Co., Gimhae, Republic of Korea), salt (Hanju, Ulsan, Republic of Korea), concentrated soy protein (Suizhong Songzhiyuan Vegetable Protein Co., Ltd., Huludao, China), xylose (ES Food, Gunpo, Republic of Korea), and phosphate complex (ES Food, Gunpo, Republic of Korea). The brine was used at 100% by weight of mackerel, and the formulation ratio is shown in Table 1.

The ingredients of the senior-friendly stewed mackerel product are shown in Table 2. It comprised 210 g (70%) of mackerel and 90 g (30%) of sauce, constituting a total weight of 300 g. To prepare the mackerel stew product, purified water (Joylife Co., Gimhae, Republic of Korea), ganjang (Daesang Corp., Seoul, Republic of Korea), sugar (Samyang Corp., Seoul, Republic of Korea), gochujang (Sajo Daerim Co., Ltd., Seoul, Republic of Korea), red pepper powder (Dr. Pepper Co., Ltd., Jeongeup, Republic of Korea), raw garlic concentrate (ES Food, Gunpo, Republic of Korea), ginger extract (Haechansol Food, Asan, Republic of Korea), calcium lactate (calcium l-lactate 5-hydrate powder; Corbion PURAC, Amsterdam, The Netherlands), sesame oil (CJ Cheiljedang Corp., Seoul, Republic of Korea), black pepper powder (Ottogi Co., Ltd., Anyang, Republic of Korea), and poly-gamma-glutamic acid (Vedan Enterprise Corp., Taichung, Taiwan) were used.

Calcium lactate is a white, tasteless, odorless substance that is highly soluble in water (5 g/100 mL). It also exhibits an optimal absorption rate in the body, with no toxicity issues [48,49]. The calcium lactate used in this study contained a pure calcium content of 13%, had no noticeable bitterness or off-flavors, and comprised a formulation ratio that was determined after several preliminary experiments, considering the dietary reference intakes for Koreans (700–800 mg for people aged >50 years) [50]. Poly-gamma-glutamic acid is a water-soluble substance derived from soybean-fermented foods, and it is not toxic to humans [51]. It also prevents phosphorus from binding to calcium in the small intestine, rendering it insoluble, and it promotes calcium absorption in the body [52]. Its RDA is 60–70 mg [53], and the formulation ratio was set accordingly.

### 2.2. Manufacture of Texture-Modified Mackerel Stew Prototypes

The sauce for the stewed mackerel was prepared by referring to a previously published recipe [54], and the ingredients included those in commercially available stewed mackerel as well as modified and supplemented proportions and cooking methods after preliminary experiments. All powdered ingredients were sieved through a 20-mesh sieve before mixing and were subsequently weighed, mixed, and heated for 3 min. The product was then sieved through a 20-mesh sieve for a second time and aged in a refrigerator (2 °C) for 24 h before use [55].

**Figure 1:**
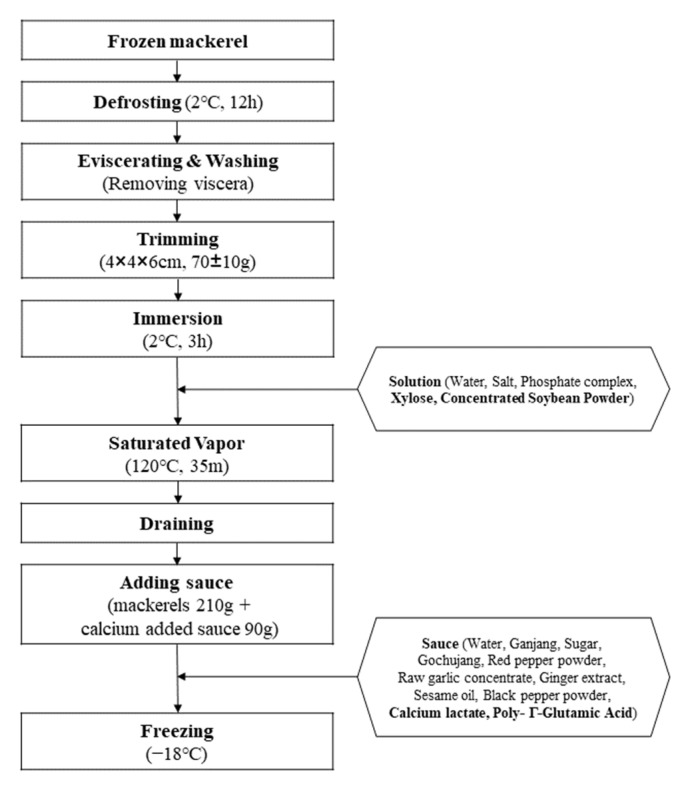
Processing flowsheet of texture-modified mackerel stew products for seniors.

The preparation of the texture-modified mackerel stew product is described in Figure 1. First, the frozen mackerel were thawed by refrigeration (2 °C) for 12 h; thereafter, the viscera were removed and washed under running water. The washed mackerel was cut into 4 × 4 × 6 cm pieces (70 ± 1 g) and immersed in brine solution at 2 °C for 3 h. The brine was prepared by mixing the ingredients listed in Table 1 in proportion and immersing the pre-treated mackerel in a 1:1 ratio to seal it. After immersion, the mackerel was separated from the brine and cooked in a saturated vapor cooker (CL-210N, Miura Co., Ltd., Tokyo, Japan) at 120 °C for 35 min. Afterwards, the water containing the oil from the cooking process was drained and discarded, and the product was packaged in a white, translucent, disposable, microwave-safe container (136 × 103 × 56 mm; Togopack, Goyang, Republic of Korea) with the previously prepared marinade in a 7:3 ratio. The prepared product was frozen (−18 °C) and used for the experiment within 5 days. Analysis of the manufactured product revealed protein and calcium contents of 21.09 g/100 g and 131.00 mg/100 g, respectively, meeting the standards for senior-friendly food in Korea [56].

### 2.3. Comparison of Consumer Acceptance of Prototypes with That of Commercial Products

#### 2.3.1. Participants

A total of 105 adults aged 50–95 years (40 aged <75 years and 65 aged ≥75 years) who had no objection to consuming fish and fish products and were not allergic to the ingredients used in the products were selected from volunteers recruited via a notice on the bulletin board of Seodaemun Senior Citizens’ Center located in Seodaemun-gu, Seoul, Republic of Korea. These individuals were capable of making decisions independently and could eat without any assistance. A total of 98 valid samples (40 for individuals aged <75 years and 58 for those aged ≥75) were used in the final analysis after excluding non-compliant or inappropriate responses. This study was approved by the Institutional Review Board of Ewha Womans University (IRB approval number: ewha-202305-0006-01). 

Generally, late adulthood is defined as those aged ≥65 years; however, we included those aged ≥50 years as “middle adulthood” (potential consumers) who will use the product in the future [57,58,59,60]. In response to the expansion of old age owing to increased life expectancy, several researchers have defined those aged 65–74 years as young–old adults and those aged ≥75 years as old–old adults based on a cutoff age of 75 years [61,62,63,64]. In addition, gustatory function gradually declines with age, with a sharp decline after the age of 75 [65], and older adults aged >75 years are significantly more likely to predominantly consume soft foods that can be consumed with the tongue [66]. Therefore, considering the differences in taste perception and chewing ability, the participants were divided into mid-to-late adulthood (age: 50–74 years) and very late adulthood (age: ≥75 years) based on a cutoff age of 75 years, and the results were compared.

#### 2.3.2. Samples and Sample Preparation

**Figure 2:**
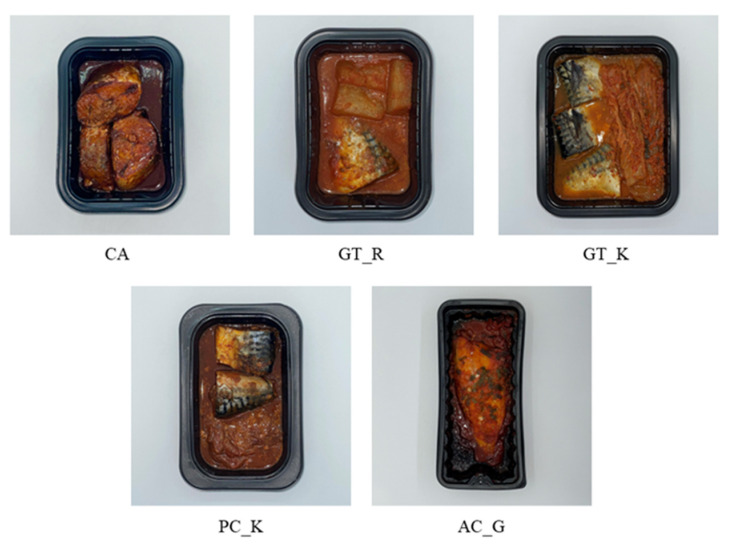
Samples of five types of mackerel stew products.

The samples used in this study are presented in Table 3, and a total of five frozen mackerel products were used as samples, including one manufactured product (calcium-added mackerel stew [CA]) and four commercial products (AC_G, GT_R, GT_K, and PC_K) (Figure 2). For the commercial mackerel products, an online shopping platform was used to prioritize microwave-heated frozen products among those with high sales volumes. In addition, considering the state of the older participants, products with fish bones softened via saturated vapor treatment (GT_R, GT_K, and PC_K) or deboned products (AC_G) were selected as the final samples. Sample hardness was measured according to the Third Act to confirm whether it met the 1st stage standard (hardness: 50,000–500,000 N/m^2^) of senior-friendly food in Korea [56].

The samples used in this study were all frozen (−18 °C) after manufacture or purchase and used within 5 days. The frozen products were heated in a microwave oven (MS23K3513AW; Samsung Electronics, Suwon, Republic of Korea) for 2–5 min using the standard cooking method for each product and served with a three-digit random number label, measuring 4 × 2 × 2 cm (10 ± 1 g) each. The samples were also sealed in a zippered bag with a hot pack to maintain temperature until evaluation, stored in a Styrofoam™ box at 50 ± 5 °C, and presented within 1 h. The samples were randomized to minimize errors arising from the order of presentation and provided with 130 g of instant rice (Hetbahn, CJ Cheiljedang Corp., Seoul, Republic of Korea) at 90 ± 2 °C and 500 mL of room temperature drinking spring water (Baeksansoo, Nongshim Co., Seoul, Republic of Korea).

#### 2.3.3. Questionnaire

The questionnaire was self-administered, and it comprised 12 questions on demographics and general information and 55 on acceptance, intensity, and consumer attitudes toward the five samples (11 questions for each sample). Upon tasting each sample, the consumers rated the overall appearance, odor, flavor, and texture likings on a standard 9-point hedonic scale (1 = extremely dislike, 5 = neither like nor dislike, and 9 = extremely like) [67,68]. To obtain realistic “liking” results on the appearance of the commercially available products, photos of the stewed mackerel products were presented to evaluate the appearance liking (Figure 2). Additionally, each sample’s hardness was evaluated on a 9-point intensity scale (9 = extremely soft and 1 = extremely hard) [20]. The reasons for (dis)liking the samples were selected using the check-all-that-apply (CATA) method. The CATA list of attributes is shown in Table 4, and it comprised terms derived by four sensory evaluators through preliminary experiments and terms from previous studies [26,55,69].

### 2.4. Statistical Analysis

All statistical analyses were performed using IBM SPSS Statistics (version 22.0; SPSS Inc., Chicago, IL, USA) and XLSTAT software (version 2021.5; Addinsoft, New York, NY, USA). Concerning the acceptance testing data, an analysis of variance (ANOVA) was performed using a generalized linear mixed (GLM) model to determine if there were significant differences in acceptance, attribute intensity, and consumer attitudes based on sample and age. The GLM model used was as follows: attributes = sample + age + (age × sample). In addition, a one-way ANOVA was conducted to determine if there were significant differences in acceptance, attribute intensity, and consumer attitudes among the samples, followed by Duncan’s multiple range test at the *p* < 0.05 level for post hoc analysis. An independent samples *t*-test was conducted to determine if there were significant differences in the overall liking, appearance liking, and familiarity in each age group by sample. 

Regarding the CATA data, the terms selected by >20% of consumers were selected for further analysis and analyzed by age group. A chi-square test was performed to check for significant differences by sample and age group, and a correspondence analysis was undertaken to visualize the correlations among the samples and reasons for (dis)liking. The correspondence analysis is a multivariate statistical analysis procedure applied to multivariate frequency data. This method extracts the key factors that explain the main variability of the data, visually summarizes the correlation between the dependent variables, and characterizes the objects using the dependent variables. This method was appropriate for the CATA data, since the data were organized as frequency data. In the sensory science field, a correspondence analysis is commonly utilized to analyze CATA data and summarize the product and attribute relationships in a 2- or 3-dimension plot [70,71,72].

Finally, to determine the effects of consumer preference, attribute intensity, and perceptions regarding purchase and recommendation intentions based on age, a multiple regression analysis was conducted with “overall liking”, “appearance liking”, “odor liking”, “flavor liking”, “texture liking”, “hardness intensity”, and “familiarity” used as independent variables and “purchase intention” and “recommendation intention” used as dependent variables.

## 3. Results

### 3.1. Demographic Characteristics

Regarding sex, 84.7% of the participants were women, and in terms of age, 53% of the participants were between 70 and 79 years of age. Regarding living status, 41.8% lived with a spouse, 29.6% lived alone, 13.3% lived with a son(s) and/or daughter(s), and 15.3% lived with a spouse, son(s), and/or daughter(s). With regards to monthly living costs, 34.7% spent less than 1 million won, while 69.4% spent less than 1.9 million won. A comparison of the mid-to-late adulthood group (50–74 years of age) with the very late adulthood group (≥75 years of age) revealed that the latter were more likely to live alone and incur monthly living expenses ≤1 million won.

The respondents’ chewing characteristics are shown in Table 5. Among the participants, 91.8% were able to chew easily, and only 11.3% reported any discomfort, exhibiting consistency with the results of Kwon (2020) [73], who found that 96.6% of their participants could chew food easily. However, considering that 76% of the participants in that study were under the age of 70, the participants in the present study, 81.6% of whom were over the age of 70, are believed to have exhibited relatively better chewing skills. No significant differences in chewing characteristics were noted between the mid-to-late adulthood group (50–74 years of age) and the very late adulthood group (≥75 years of age) in this study; nevertheless, 15.5% of the very late aged group were at least slightly uncomfortable. This proportion exceeded the 5% of the mid-to-late aged group. This was considerably lower than the proportion yielded by the Ministry of Health and Welfare’s survey of seniors aged ≥65 years, which found that 39.5% of seniors were uncomfortable, while 34.8% and 4.7% were somewhat and very uncomfortable, respectively. The survey also found that the older the age, the higher the rate of chewing discomfort [74].

### 3.2. Effects of Product and Age on Consumer Acceptance and Perception Ratings

The influence of sample, age, and sample × sample interaction effects on the liking and intensity ratings of and consumer attitudes toward the mackerel stew product samples analyzed using the GLM model are shown in Table 6. Significant differences in overall liking, photograph-based appearance liking, appearance liking, odor liking, flavor liking, texture liking, hardness intensity, purchase intention, recommendation intention (*p* < 0.001), and familiarity (*p* < 0.05) were found among the samples. The model was not significant for age group (Wilk’s lambda = 0.477); however, a significant difference was noted in purchase intention by age (*p* < 0.05). Finally, the interaction between sample and age group was not significant (Wilk’s lambda = 0.604); nonetheless, significant differences were observed in odor liking, texture liking (*p* < 0.01), overall liking, appearance liking, taste liking, familiarity, and purchase intention (*p* < 0.05). The CA sample received the highest scores for overall liking, photograph-based appearance liking, appearance liking, odor liking, taste liking, flavor liking, texture liking, firmness, purchase intention, recommendation intention (*p* < 0.001), and familiarity (*p* < 0.05). 

Regarding purchase intention, which exhibited age-related differences, the very late adulthood group rated higher (5.51 ± 1.93) than the mid-to-late adulthood group (5.16 ± 2.16). In addition, significant interactions between sample and age (Sample × Age) were found for seven items (overall liking, appearance liking, odor liking, flavor liking, texture liking, familiarity, and purchase intention), suggesting sample-dependent differences in the evaluations across each age group. An independent samples *t*-test was conducted to check for significant differences between age groups by sample for these items, and the results are presented in Table 7. The CA, GT_K, and PC_K samples did not display significant differences by item; nevertheless, the PC_K sample received higher ratings from the mid-to-late adulthood group. The GT_R sample drew higher ratings for all the items from the mid-to-late adulthood group, with significant differences in appearance liking and familiarity (*p* < 0.05). In contrast, the AC_G sample attracted higher ratings from the very late adulthood group, with significant differences in overall liking, appearance liking, and familiarity (*p* < 0.05), as well as in odor liking, flavor liking, texture liking, and purchase intention (*p* < 0.01).

### 3.3. Comparison of Factors Affecting Consumer Attitudes by Age

A multiple regression analysis was conducted to determine the effects of acceptance, attribute intensity, and consumer attitude on purchase and recommendation intentions across age groups, with “overall liking”, “appearance liking”, “odor liking”, “flavor liking”, “texture liking”, “hardness intensity”, and “familiarity” used as independent variables. First, a purchase intention analysis revealed that flavor liking, texture liking, and familiarity had a significant impact on purchase intention in the mid-to-late adulthood group (Table 8). In the very late adulthood group, flavor liking, familiarity, and odor liking were found to have a significant impact on purchase intention (Table 9). Thus, the higher the flavor liking and familiarity ratings, the greater the purchase intention, regardless of age group.

A recommendation intention analysis revealed that flavor liking, overall liking, and familiarity, in this order, had a significant effect on recommendation intention in the mid-to-late adulthood group (Table 10). In the very late adulthood group, flavor liking, familiarity, overall liking, and hardness intensity had a significant effect on recommendation intention (Table 11). Thus, regardless of age group, the higher the flavor liking, overall-liking, and familiarity ratings, the greater the recommendation intention.

### 3.4. Comparison of Reasons for (Dis)liking by Age

To identify the factors inducing consumer acceptance of senior-friendly stewed mackerel products by age, the preferred and non-preferred factors were analyzed in the overall study population and in each age group. Regarding the “liking” reasons, members of the mid-to-late adulthood group selected 6.1 words per sample, while those of the very late adulthood group selected 4.7 words. In terms of the “disliking” reasons, they selected 6.0 and 4.1 words per sample, respectively. “Mackerel softness”, “mackerel moistness”, “good for health”, “not heavy”, “light”, and “ganjang odor/taste” were frequently selected as liking factors for the five different types of stewed mackerel, while “artificial”, “not harmonious”, and “greasy” were frequently selected as disliking factors.

**Figure 3:**
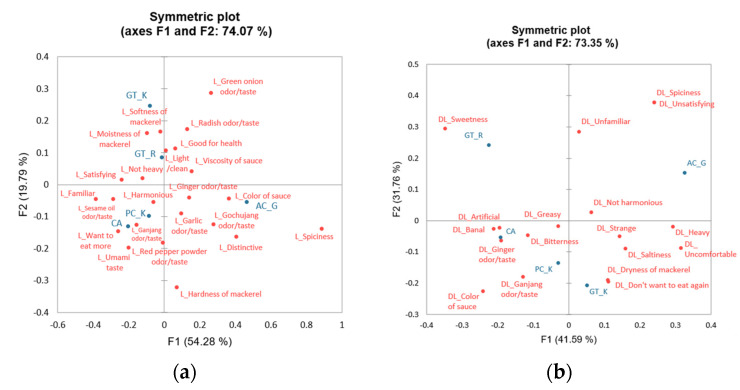
Correspondence analysis plot of (**a**) reasons for liking attributes and their corresponding sample loadings and (**b**) reasons for disliking attributes and their sample loadings in the 50–74-year age group.

In the mid-to-late adulthood group, the four samples (GT_R, GT_K, PC_K, and CA) were liked owing to familiar and satisfying features (Figure 3). Among them, the GT_K and GT_R samples were liked because of their green onion odor/taste, odor/taste, mackerel moistness, and mackerel softness. In addition, the PC_K and CA samples tended to be liked due to umami, sesame oil odor/taste, ganjang odor/taste, chili powder flavor/flavor, and “want to eat more” characteristics. Spiciness and gochujang odor/taste, sauce color, mackerel firmness, and distinctiveness were considered reasons for liking the AC_G sample. 

In the correspondence analysis plot of the disliking attributes, the GT_R, CA, and PC_K samples were disliked for their ginger odor/taste and artificial and banal features. Among them, the GT_R sample was disliked for its strong sweetness and unfamiliarity, while the PC_K sample was disliked for its ganjang odor/taste and sauce color. The AC_G and GT_K samples tended to be disliked for their heavy and uncomfortable characteristics, especially the spiciness and unfamiliar and unsatisfying characteristics of the AC_G sample, as well as the sauce color, mackerel dryness, and “lack of desire to eat again” for the GT_K sample.

**Figure 4:**
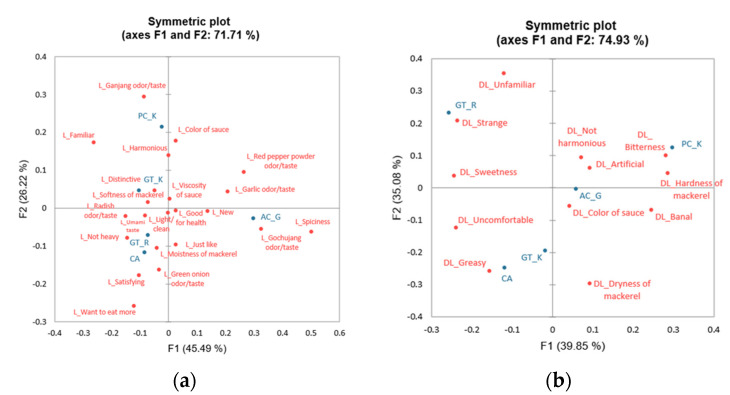
Correspondence analysis plot of (**a**) reasons for liking attributes and their corresponding sample loadings and (**b**) reasons for disliking attributes and their sample loadings in the ≥75-year age group.

In the very late adulthood group, the participants tended to like the PC_K, GT_K, GT_R, and CA samples owing to their umami, radish odor/taste, mackerel softness, distinctive, and “not heavy” characteristics (Figure 4). Among them, the PC_K sample was preferred because of its ganjang odor/taste, sauce color, familiarity, and harmonious characteristics. In addition, the CA sample tended to be preferred, owing to its green onion odor/taste, mackerel moistness, satisfying, and “wanting more” characteristics. The AC_G sample appeared to be liked for its spicy, red pepper powder odor/taste, garlic odor/taste, gochujang odor/taste, and new attributes. 

In the correspondence analysis plot of the disliking attributes, the GT_R sample tended to be disliked because of its sweetness and unfamiliar and strange feel. In addition, the GT_K and CA samples were considered to be disliked owing to their unpleasant and unfamiliar characteristics and mackerel dryness. The AC_G and PC_K samples were disliked because of their artificial and inharmonious characteristics and mackerel hardness, and among them, bitterness and banal features were identified as reasons for disliking the PC_K sample.

## 4. Discussion

This study aimed to develop a senior-friendly food product that caters to the physical characteristics and preferences of older consumers. Mackerel was selected as an animal protein source for the older population, and the product was prepared as a stew using a moist-heat cooking method preferred by older adults. In addition, marination and saturated vapor treatment, a type of high-pressure processing, were used to soften the mackerel. High-pressure processing is used to manufacture texture-modified foods for older adults because it can soften food while retaining flavor and nutrients, and it potentially improves bioavailability [75]. Further, the senior-friendly stewed mackerel product was manufactured using a sauce containing calcium, the deficiency of which is a cause for concern, and poly-gamma-glutamic acid, which increases the rate of calcium absorption in the body. The product’s marketability was confirmed by comparing it with four commercial products via a consumer survey. 

Among the demographic characteristics, living status and monthly living expenses were significantly different between the mid-to-late adulthood group (50–74 years) and the very late adulthood group (≥75 years) (*p* < 0.01). In addition, while approximately 50% of older adults generally experience mastication difficulties [76], the proportion of participants with chewing problems in this study was low despite their age. This may be explained by the fact that the study participants were voluntarily recruited from welfare centers; therefore, healthy and active seniors were likely to have participated in this study.

### 4.1. Effects of Product and Age on Consumer Acceptance and Attitudes

Acceptance tests of the five types of stewed mackerel revealed that the developed CA sample was the most preferred, and the ratings of all the items were significantly higher, confirming its commercialization potential. An age-related difference in purchase intention was noted, with the very late adulthood group scoring higher (*p* < 0.05). In addition, sample–age group interactions exhibited significant differences in odor liking, texture liking (*p* < 0.01), overall liking, appearance liking, flavor liking, familiarity, and purchase intention (*p* < 0.05). Moreover, higher flavor-liking and familiarity ratings increased purchase intention, and higher flavor-liking, overall-liking, and familiarity ratings increased recommendation intention. The “fishy” off-flavors of fish products can be offensive to many consumers; thus, monitoring fishy flavors during product development is important [77]. This study also confirmed that flavor is an important factor in food for older adults. Meanwhile, in a study of older adults, liking increased as familiarity with food odors increased, suggesting the importance of considering familiarity in senior-friendly foods [78].

### 4.2. Comparison of Factors Affecting Consumer Attitudes by Age

Overall, no age-related differences in acceptance were noted in the CA and GT_K samples; however, the GT_R and PC_K samples were preferred by the mid-to-late aged adults, whereas the AC_G sample was preferred by the very old adults. The mid-to-late adulthood group liked the GT_R sample, owing to its green onion odor/taste, radish odor/taste, mackerel moistness, and mackerel softness; nevertheless, the very late adulthood group did not like it because of its sweetness and unfamiliarity. In addition, the mid-to-late aged adults selected umami, sesame oil odor/taste, ganjang odor/taste, red pepper powder odor/taste, “want to eat more”, familiarity, and satisfying characteristics as reasons for liking the PC_K sample, while the very old adults selected bitterness and a banal taste as disliking reasons. The people in very late adulthood selected spiciness, red pepper powder odor/taste, garlic odor/taste, gochujang odor/taste, and newness as reasons for liking the AC_G sample, which displayed significantly higher liking ratings with increasing age (*p* < 0.05), while the middle- and late-aged people selected spiciness, unfamiliarity, unsatisfying, heavy, and discomfort as reasons for disliking this sample. In particular, the AC_G sample analysis suggested that the very old adults had a relatively greater preference for spicy flavors than the mid-to-late-aged adults. 

In this study, a senior-friendly food product was manufactured using mackerel and was found to have protein and calcium contents of 21.09 g/100 g and 131.00 mg/100 g, respectively, thus meeting the standards for senior-friendly foods according to Korean industrial standards. Therefore, it is expected to provide basic data for research on fish- and mackerel-based senior-friendly foods, which is currently in its initial stages. Moreover, by adding calcium to a product that can be easily consumed as a home meal, the possibility of providing additional nutrients that are often lacking in older adults was confirmed. In addition, consumer acceptance of the developed product was significantly greater than that of high-volume products on the market, and nutritional components that are vulnerable in older adults, including calcium, could be actively applied. Finally, “baby boomers”, who are expected to become the largest consumer group in the senior market [79], were included in the survey. By analyzing the survey results according to age, the product is believed to be potentially useful as a basis for developing food concepts and product ideation for the next generation of seniors.

However, this survey was limited to urban residents; predominantly women. The area of residence is reportedly related to income and food consumption patterns [80,81,82]; nonetheless, this study did not include older people in small and medium-sized cities and rural areas, thus potentially limiting the interpretation of the results. Future studies reflecting urban–rural comparisons and income level are warranted. Soy protein concentrates, the softening material used in this study, have the potential to be problematic for consumers with soy allergies. Therefore, it is essential to clearly label the developed product to indicate that it contains soy if it is commercialized.

This study was conducted among adults over 50, and mostly in adults over 70. There is an obvious need to conduct sensory and consumer studies with older adults, as their perception and food acceptance cannot be extrapolated from data collected in an adult population due to differences related to generational and age effects. Despite this, older adults have limited attentional abilities and tend to give higher hedonic scales than adults. To obtain more accurate results for senior-friendly foods, a panel of older adults with the same characteristics as the product’s target audience should be trained and interviewed over time [83]. Additionally, difficulties in chewing can lead to food choices to avoid foods that are hard to eat and may result in reduced nutrient intake, including lower protein consumption [84]. Likewise, food pickiness among older adults could constrain their dietary options, with 23% of them being reported as picky eaters [85]. Also, fish is a polarizing food, so an individual’s typical preference can influence the acceptance of the product. Hence, it is essential to conduct product development and consumer preference studies using a variety of animal protein sources, including meat and poultry.

## 5. Conclusions

This study developed a protein-rich food product by processing mackerel with marination and saturated vapor treatment. This food can be utilized in the senior-friendly food industry, which still lacks product diversity. Additionally, this study and confirmed its marketability based on a high acceptance. This study also confirmed that flavor liking and familiarity are essential to consumer attitudes toward senior-friendly foods. It serves as reference data for the development of diverse fish- and mackerel-based foods for older adults, especially those with chewing difficulties.

## Figures and Tables

**Table 1:** Formula for preparing the immersion solution.

Ingredients	Manufacturing Company	g	%
Water	Joylife Co., Gimhae, Republic of Korea	194.25	92.5
Salt	Hanju, Ulsan, Republic of Korea	6.3	3
Concentrated soybean powder	Suizhong Songzhiyuan Vegetable Protein Co., Ltd., Huludao, China	4.2	2
Xylose	ES Food, Gunpo, Republic of Korea	3.15	1.5
Phosphate complex	ES Food, Gunpo, Republic of Korea	2.1	1
Total		210	100

**Table 2:** Formula for preparing texture-modified mackerel stew products for seniors.

Ingredients	Manufacturing Company	g	%
**Norwegian mackerel**	Sang Won Fisheries, Hanam, Republic of Korea	210	70
**Calcium-added sauce**			
Water	Joylife Co., Gimhae, Republic of Korea	60	20.00
Ganjang	Daesang Corp., Seoul, Republic of Korea	10.5	3.50
Sugar	Samyang Corp., Seoul, Republic of Korea	6.3	2.10
Gochujang	Sajo Daerim Co., Ltd., Seoul, Republic of Korea	5.1	1.70
Red pepper powder	Dr.Pepper Co., Ltd., Jeongeup, Republic of Korea	4.05	1.35
Raw garlic concentrate	ES Food, Gunpo, Republic of Korea	1.8	0.60
Ginger extract	Haechansol Food, Asan, Republic of Korea	0.9	0.30
Calcium lactate	Corbion PURAC, Amsterdam, The Netherlands	0.63	0.21
Sesame oil	CJ Cheiljedang Corp., Seoul, Republic of Korea	0.51	0.17
Black pepper powder	Ottogi Co., Ltd., Anyang, Republic of Korea	0.15	0.05
Poly-gamma-glutamic acid	Vedan Enterprise Corp., Taichung, Taiwan	0.06	0.02
Total		300	100

**Table 3:** Mackerel stew product sample information and standard recipes.

Sample Code ^1^	Product Name	Manufacturer	Main Ingredients	Standard Recipes	Certification ^2^
CA	Calcium-added mackerel stew	Manufactured by the researcher			
GT_R	[Greating] Mackerel stew with edible bones	Hyundai Green Food Co., Seongnam, Republic of Korea	Mackerel (*Scomber scombrus*) 33.1%, Radish 32.3%, garlic 1.4%, and green onion 1.4%	2–3 min in the microwave	KS 1 (Able to chew)
GT_K	[Greating] Mackerel kimchi stew with edible bones	Hyundai Green Food Co., Seongnam, Republic of Korea	Kimchi 42.8% and mackerel (*Scomber scombrus*) 35.8%	3–3.5 min in the microwave	None
PC_K	[Pul’s care] Mackerel kimchi stew	Farming Corporation, Hanguksigyeon Co., Iksan, Republic of Korea	Mackerel (*Scomber scombrus*) 52.2% and kimchi 34.8%	2.5–3 min in the microwave	None
AC_G	[And Cook] Boneless mackerel stew	Deungpureunsikpum Co., Busan, Republic of Korea	Mackerel (*Scomber japonicus*) and green onion	5 min in the microwave	None

^1^ Each sample code was formulated based on the product’s brand name and main ingredients. ^2^ Certification as an excellent Korean senior-friendly food.

**Table 4:** CATA list of “liking” and “disliking” terms for stewed mackerel samples.

	Liking Terms (n = 32)	Disliking Terms (n = 33)
Sensory factors Attribute factors	Sweetness	Sweetness
	Saltiness	Saltiness
	Bitterness	Bitterness
	Umami taste	Umami taste
	Spiciness	Spiciness
	Gochujang odor/taste	Gochujang odor/taste
	Red pepper powder odor/taste	Red pepper powder odor/taste
	Ganjang odor/taste	Ganjang odor/taste
	Garlic odor/taste	Garlic odor/taste
	Ginger odor/taste	Ginger odor/taste
	Sesame oil odor/taste	Sesame oil odor/taste
	Radish odor/taste	Radish odor/taste
	Kimchi odor/taste	Kimchi odor/taste
	Green onion odor/taste	Green onion odor/taste
	Sauce color	Sauce color
	Sauce viscosity	Sauce viscosity
	Mackerel softness	Mackerel softness
	Mackerel hardness	Mackerel hardness
	Mackerel moistness	Mackerel dryness
Non-sensory factors	Just like	Just do not like
	Familiar	Banal
	New	Strange
	Distinctive	Unfamiliar
	Stimulating	Stimulating
	Gourmet	Artificial
	Good for health	Not healthy
	Feel better	Feel worse
	Want to eat more	Don’t want to eat again
	Not heavy	Heavy
	Satisfying	Unsatisfying
	Light/clean	Uncomfortable
	Harmonious	Not harmonious
		Greasy

**Table 5:** Participants’ general characteristics (n = 98) ^1^.

	Total (n = 98)	50–74 Years (n = 40)	≥75 Years (n = 58)	*p*-Value ^2^
Chewing ability				0.637
Able to chew easily	90 (91.8)	38 (95.0)	52 (89.7)	
Able to mash with gums	4 (4.1)	1 (2.5)	3 (5.2)	
Able to swallow without chewing				
Chewing difficulty				0.388
Very uncomfortable	3 (3.1)	0 (0)	3 (5.2)	
Somewhat uncomfortable	8 (8.2)	2 (5.0)	6 (10.3)	
Neutral	14 (14.3)	7 (17.5)	7 (12.1)	
Somewhat comfortable	37 (37.8)	14 (35.0)	23 (39.7)	
Comfortable	36 (36.7)	17 (42.5)	19 (32.8)	

^1^ Values are presented as frequencies (percentages). ^2^ *p*-values for differences in distribution between the two groups were tested using a χ^2^ test for categorical variables.

**Table 6:** Effects of sample and age on the acceptance, sensory properties, and consumer attitudes toward the mackerel stew product samples.

	Sample		Age		Sample × Age	
	*F*-Value	*p*-Value	*F*-Value	*p*-Value	*F*-Value	*p*-Value
Overall liking	12.804	<0.001	0.000	0.989	2.897	<0.05
Appearance liking_pic ^1^	6.497	<0.001	0.834	0.362	0.729	0.572
Appearance liking_spl	6.904	<0.001	0.404	0.525	3.364	<0.05
Odor liking	8.289	<0.001	0.351	0.554	4.021	<0.01
Flavor liking	8.326	<0.001	0.423	0.516	2.706	<0.05
Texture liking	11.577	<0.001	0.051	0.822	3.936	<0.01
Hardness intensity	5.790	<0.001	0.132	0.717	1.517	0.196
Degree of familiarity	2.691	<0.05	0.081	0.776	2.721	<0.05
Purchase intention	8.888	<0.001	4.010	<0.05	2.920	<0.05
Recommendation Intention	8.473	<0.001	3.432	0.065	1.764	0.135

^1^ Photographs of the products in practice were presented to the participants to evaluate appearance liking. Thereafter, appearance liking was evaluated again based on the actual sample during sensory evaluation.

**Table 7:** Differences according to age classification and sample ^1,2^.

	Total (n = 98)	50–74 Years (n = 40)	≥75 Years (n = 58)	*p*-Value ^3^
**CA**				
Overall liking	7.02 ± 1.55	7.03 ± 1.54	7.02 ± 1.56	0.981
Appearance liking	6.71 ± 1.47	6.78 ± 1.61	6.67 ± 1.38	0.736
Odor liking	6.50 ± 1.71	6.45 ± 1.72	6.53 ± 1.71	0.811
Flavor liking	6.83 ± 1.71	6.58 ± 1.99	7.00 ± 1.47	0.253
Texture liking	6.85 ± 1.66	6.83 ± 1.81	6.86 ± 1.57	0.914
Degree of familiarity	5.84 ± 1.93	5.90 ± 1.98	5.79 ± 1.90	0.789
Purchase intention	6.41 ± 1.67	6.15 ± 1.96	6.59 ± 1.43	0.232
**GT_R**				
Overall liking	5.66 ± 1.99	6.05 ± 2.21	5.40 ± 1.80	0.125
Appearance liking	5.88 ± 1.84	6.33 ± 1.82	5.57 ± 1.81	<0.05
Odor liking	5.32 ± 1.94	5.65 ± 1.86	5.09 ± 1.97	0.157
Flavor liking	5.54 ± 1.99	5.85 ± 1.92	5.33 ± 2.02	0.202
Texture liking	5.94 ± 1.92	6.25 ± 1.75	5.72 ± 2.01	0.183
Degree of familiarity	5.11 ± 2.06	5.63 ± 1.93	4.83 ± 1.90	<0.05
Purchase intention	5.33 ± 2.13	5.30 ± 2.02	4.98 ± 2.10	0.457
**GT_K**				
Overall liking	5.50 ± 1.94	5.43 ± 2.15	5.55 ± 1.80	0.752
Appearance liking	5.56 ± 1.95	5.53 ± 2.01	5.59 ± 1.92	0.879
Odor liking	5.43 ± 1.89	5.33 ± 2.06	5.50 ± 1.78	0.654
Flavor liking	5.54 ± 1.94	5.58 ± 2.10	5.52 ± 1.85	0.886
Texture liking	5.89 ± 1.89	5.95 ± 1.92	5.84 ± 1.88	0.788
Degree of familiarity	5.04 ± 2.13	5.10 ± 1.86	5.36 ± 2.01	0.516
Purchase intention	5.06 ± 2.27	4.78 ± 2.19	5.22 ± 2.09	0.307
**PC_K**				
Overall liking	5.72 ± 2.08	6.05 ± 2.23	5.50 ± 1.95	0.199
Appearance liking	5.82 ± 1.90	6.20 ± 1.87	5.55 ± 1.89	0.097
Odor liking	5.78 ± 1.82	6.08 ± 1.90	5.57 ± 1.75	0.177
Flavor liking	5.74 ± 1.96	5.95 ± 2.29	5.60 ± 1.70	0.417
Texture liking	6.11 ± 1.80	6.43 ± 1.81	5.90 ± 1.77	0.154
Degree of familiarity	5.37 ± 1.94	5.43 ± 2.11	5.33 ± 1.83	0.808
Purchase intention	5.27 ± 1.91	5.38 ± 2.10	5.19 ± 1.79	0.640
**AC_G**				
Overall liking	5.23 ± 2.16	4.60 ± 2.04	5.67 ± 2.14	<0.05
Appearance liking	5.62 ± 1.80	5.08 ± 1.83	6.00 ± 1.69	<0.05
Odor liking	5.22 ± 1.90	4.45 ± 1.68	5.76 ± 1.87	<0.01
Flavor liking	5.46 ± 1.99	4.83 ± 2.02	5.90 ± 1.85	<0.01
Texture liking	5.15 ± 2.09	4.38 ± 1.98	5.69 ± 2.00	<0.01
Degree of familiarity	5.06 ± 1.90	4.48 ± 2.03	5.47 ± 1.71	<0.05
Purchase intention	5.01 ± 2.05	4.18 ± 2.10	5.59 ± 1.82	<0.01

^1^ Values are expressed as the mean ± standard deviation. ^2^ 9-point Likert scale: 9 = highest, 1 = lowest. ^3^ *p*-values for mean differences between the two groups were tested using an independent samples *t*-test for continuous variables.

**Table 8:** Effects of liking factors and degree of familiarity on purchase intention in the 50–74-year age group (n = 40).

Dependent Variable	Independent Variable	Unstandardized Coefficients (B)	Standardized Coefficients (β)	*t*	*p*-Value
Purchase intention	(Constant)	−0.098		−0.303	0.763
	Flavor liking	0.367	0.362	4.983	<0.001
	Texture liking	0.317	0.298	4.102	<0.001
	Degree of familiarity	0.235	0.221	3.868	<0.001
R^2^ = 0.602, adjusted R^2^ = 0.596, F = 98.942, *p* = 0.000					

**Table 9:** Effects of liking factors and degree of familiarity on purchase intention in the ≥75-year age group (n = 58).

Dependent Variable	Independent Variable	Unstandardized Coefficients (B)	Standardized Coefficients (β)	*t*	*p*-Value
Purchase intention	(Constant)	0.601		1.867	0.063
	Flavor liking	0.345	0.334	4.822	<0.001
	Degree of familiarity	0.307	0.299	6.456	<0.001
	Odor liking	0.219	0.211	3.085	<0.01
R^2^ = 0.468, adjusted R^2^ = 0.462, F = 83.834, *p* = 0.000					

**Table 10:** Effects of liking factors and degree of familiarity on recommendation intention in the 50–74-year age group (n = 40).

Dependent Variable	Independent Variable	Unstandardized Coefficients (B)	Standardized Coefficients (β)	*t*	*p*-Value
Recommendation intention	(Constant)	0.092		0.280	0.780
	Flavor liking	0.423	0.398	4.729	<0.001
	Overall liking	0.285	0.275	3.167	<0.01
	Degree of familiarity	0.195	0.175	2.974	<0.01
R^2^ = 0.587, adjusted R^2^ = 0.581, F = 93.029, *p* = 0.000					

**Table 11:** Effects of liking factors and degree of familiarity on recommendation intention in the ≥75-year age group (n = 58).

Dependent variable	Independent Variable	Unstandardized Coefficients (B)	Standardized Coefficients (β)	*t*	*p*-Value
Recommendation intention	(Constant)	0.133		0.312	0.755
	Flavor liking	0.284	0.263	3.453	<0.01
	Degree of familiarity	0.263	0.246	5.010	<0.001
	Overall liking	0.238	0.229	3.037	<0.01
	Hardness intensity	0.154	0.114	2.272	<0.05
R^2^ = 0.440, adjusted R^2^ = 0.432, F = 56.054, *p* = 0.000					

## Data Availability

Data is contained within the article.
